# How the LiveWell Dorset lifestyle behaviour change service influences older adults’ physical activity behaviour: A generic qualitative study

**DOI:** 10.1016/j.puhip.2021.100164

**Published:** 2021-07-17

**Authors:** Andrew J. Powell, Sarah Thomas

**Affiliations:** Bournemouth University Clinical Research Unit, Department of Medical Sciences and Public Health, 10 St Paul's Lane, Bournemouth, Dorset, BH8 8AJ, UK

**Keywords:** Physical activity, Older adults, Behaviour change, Public health, Behaviour change technique

## Abstract

**Objectives:**

There is uncertainty around the most effective characteristics and components of interventions to increase older adults' physical activity (PA) levels. This study aimed to explore how LiveWell Dorset (LWD), a lifestyle behaviour change support service based in Dorset, South-West England, influences older adults’ PA behaviour.

**Study design:**

A qualitative study using a generic approach.

**Methods:**

Semi-structured telephone interviews were held with 12 individuals aged 55 to 65 who had accessed LWD's support to increase their PA levels, to gather their views and experiences of the service. A thematic analysis of the interview data was conducted using a hybrid approach that incorporated both inductive and deductive processes. Additionally, interview content was coded for behaviour change techniques using the Behaviour Change Technique Taxonomy version 1.

**Results:**

A number of key factors were identified that highlighted how the LWD service influences older adults’ PA behaviour. These included using a broad range of promotional and behaviour change strategies to facilitate initial engagement, providing opportunities for service users to receive social support from both professionals and peers, and emphasising person-centredness and empathy in interactions with service users.

**Conclusions:**

The findings provide valuable information regarding the needs and preferences of older adults when attempting to influence their PA behaviour, and on the reportedly helpful components of interventions that aim to do so. The findings also support the need for future research into previously highlighted topics of interest such as the roles of systemic and contextual factors and professional empathy on PA intervention engagement and outcomes.

## 1 Introduction

Physical inactivity is a significant risk factor for the development of age-related ill health and long-term disease [1]. There is a wealth of evidence suggesting that participating in regular physical activity (PA) provides a multitude of preventative health and quality of life benefits for individuals as they reach middle-age and beyond. The improvements in cardiorespiratory fitness, muscle strength, balance and mobility that result from PA participation are associated with a lower risk of cardiovascular disease, depression, falls, muscle and bone loss and cognitive decline in older adults, along with improved emotional, social, cognitive and physical functioning [2,3]. World Health Organisation guidelines recommend that to obtain these benefits, 55–64 year olds should perform at least 300 minutes of moderate intensity PA per week, and over 65s at least 150 minutes [4]. However, at present, only around 60% of over 55s in the UK are considered physically active [5]. In response to this, increasing the PA levels of older adults has become a priority for public health interventions in the UK, in order to promote healthy ageing and reduce the risk of preventable health conditions developing [6].

Despite challenges in synthesising evidence from heterogeneous studies that used predominantly randomised controlled trial designs, the most recent umbrella systematic review confirmed the effectiveness of multi-modal and multi-component PA interventions for increasing the self-reported and objectively measured PA levels of community-dwelling older adults [7]. The interventions the review examined had been implemented across a range of community settings, delivered both face-to-face and remotely by a range of professionals (e.g. GPs, nurses, occupational therapists, fitness instructors, PA coaches), involved numerous modes of PA (e.g. walking, aquatic exercise, dance), and most commonly were of a three to 12 month duration. In particular, client-centred and personalised interventions involving tailored professional guidance and ongoing support were reported to lead to improved PA participation. However, the review concluded that there was general uncertainty around the most effective intervention characteristics and components to increase older adults' PA levels, as rarely were any consistently associated with positive or negative outcomes. It is therefore imperative that research continues to explore how different interventions influence older adults’ PA behaviour, and to attempt to identify their most important features.

**Fig. 1:**
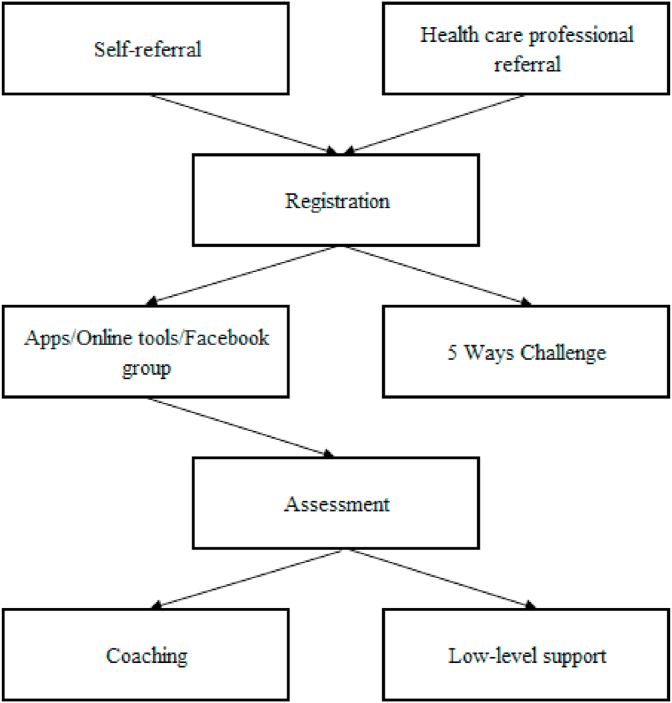
LiveWell Dorset service pathway.

The LiveWell Dorset (LWD) integrated lifestyle service, based in Dorset, South-West England, is an intervention that offers behaviour change support online and via telephone to adults in the Dorset area, with the aim of helping them to meet the Government's recommended guidelines for PA, to reach and maintain a healthy weight, to keep within the recommended limits for alcohol consumption and to stop smoking. As part of Dorset's 2016/17 Sustainability and Transformation Plan, the LWD service was developed by its operators Public Health Dorset to offer support across these four lifestyle behaviours, instead of the traditional single pathway approach, in order to maximise the efficiency, scale, reach and impact of behaviour change support in the area with minimal increased cost [8]. LWD's design followed consultation with local residents regarding their needs and views on existing services, and drew upon behaviour change literature. Consequently, the guiding principle that came to underpin the design of the LWD service was that identifying an individual's barriers to change and selecting the behaviour change techniques to overcome them would most likely lead to positive results [9]. The LWD service pathway is depicted in Fig. 1. Individuals can self-refer to LWD, and there is also a facility for health care professionals to refer their patients. Upon registration with LWD, individuals undergo an assessment, where a ‘behavioural diagnosis’ is made regarding the factors most influencing their behaviour, and the lifestyle area to focus on. Furthermore, a collaborative discussion is held regarding the level of support they require to move forward, based on the current behavioural barriers that are contributing to difficulties in making or sustaining change. For some individuals, a low level of telephone and email follow-up support and signposting is then provided by LWD for up to 12 months to facilitate the process of behaviour change and the accessing of other local services. For others, four to six sessions of more intensive telephone coaching support is delivered. LWD also offers a wrap-around digital support interface consisting of a range of apps and online tools such as a local activity finder and a calorie calculator, as well as a private LWD Facebook group, which individuals can access once they have registered. LWD also launched the ‘Five Ways Challenge’ in 2020, an online programme developed to provide weekly group email support to help individuals to improve their wellbeing and cope during the COVID-19 pandemic by focusing on daily PA, as well as social connection, awareness, learning new skills and giving to others [8].

The aim of this study was to explore how the LWD service influences PA behaviour in older adults.

## 2 Methods

### 2.1 Study Design

A qualitative study involving semi-structured telephone interviews was conducted. Ethical approval was obtained from Bournemouth University (ref. 28034).

### 2.2 Participants and recruitment

Based on resource availability, the aim was to conduct 12 telephone interviews with a convenience sample of individuals aged 55 and over who had accessed LWD's support to increase their PA levels, to gather their views and experiences of the service.

Potential participants were identified via LWD, who acted as the recruitment gatekeeper. LWD posted an advertisement on the LWD Facebook page seeking individuals aged 55 and over who had accessed the support of the service to become more active and were willing to take part in a telephone interview. Those interested in participating were asked to fill out a web form to provide their email and telephone contact details along with permission for LWD to share these details with the study researcher (AJP) to enable contact to be made. LWD passed on the details of individuals who completed the form to AJP on a first come, first served basis, and AJP co-ordinated all further study activities. AJP called potential participants to explain the purpose of the study and answer any questions that they had. If they were willing to participate, a telephone interview was arranged for a date and time convenient to them.

### 2.3 Interview procedures

The telephone interviews were conducted by AJP. Participants were sent an information sheet and a copy of the consent form via email several days prior to the telephone interview. On the day of the interview, AJP answered any questions that participants had about either form, and explained the consent process. If they agreed to take part, their verbal consent was recorded prior to the start of the interview.

For the interviews, a generic qualitative approach was employed. Generic qualitative research seeks to discover and understand people's subjective opinions and reflections of things in the outer world [10]. It, “focuses on descriptions of what people experience” [11], “simply seeks to understand a phenomenon, a process, or the perspectives and worldviews of the people involved” [12], and emphasises people's perceptions and feelings rather than the ‘meanings’ that might underlie them [13].

A topic guide was used during interviews to ensure key areas of interest were covered, while still allowing for flexibility and the possibility to follow up on unprompted content. The process of developing the topic guide involved a series of face-to-face discussions between LWD and AJP about the LWD service and its operations, as well as the examination of LWD ‘standard operating procedures’ documents by AJP. As summarised in Table 1, the topic guide contained questions about the support that participants had accessed through LWD, how LWD had facilitated their initial engagement with the service, and the outcomes of their LWD experiences. Furthermore, to gain a rounded understanding of LWD's influence on PA behaviour, questions relating to participants' previous PA backgrounds were also included. The telephone interviews were audio-recorded using a digital audio-recorder directly connected to the telephone and transcribed verbatim.

**Table 1:** Summary of the interview topic guide.

Topic Area
Previous lifestyle and physical activity habits
Previous barriers to physical activity
Source of initial awareness of LiveWell Dorset
Initial hopes/goals/expectations
Views about registration and assessment processes
Views about interventions and support received
Personal achievements/accomplishments/changes
Suggestions for improvements

### 2.4 Analysis

A thematic analysis was conducted by AJP [14], using a hybrid approach that incorporated both inductive and deductive processes [15]. Before initial familiarisation with the interview transcripts took place, a priori codes were first added to a codebook in Microsoft Word [16], largely drawn from the original interview topic guide. Then, following familiarisation, important patterns and common threads were searched for in the interview data, at which point emerging posteriori codes were added to the codebook. Once all codes had been added, they were discussed in a debrief session with the co-author (ST). The list of a priori and posteriori codes were then merged into themes, which were subsequently vetted by ST. Illustrative quotations relating to each theme were then indexed and collated, ready for summarising, reporting and interpretation.

Furthermore, to provide an additional layer of information on how LWD influences PA behaviour, the interview content was coded by AJP for behaviour change techniques (BCTs) using the BCT Taxonomy version 1 (BCTTv1) [17,18]. BCTs were coded if they pertained to components of LWD that attempted to facilitate participants' progression through the service or influence their PA behaviour. Using the BCTTv1, BCTs were identified within participants’ narratives on the basis of being observable, replicable and irreducible. All BCT labels were checked by ST, with disagreements on any labels resolved by discussion and consensus. Both authors had undertaken online training in BCT coding prior to the labelling process.

## 3 Results

### 3.1 Overview

In total, telephone interviews with 12 individuals aged 55 to 65 who had previously accessed LWD for support with increasing their PA levels were conducted. Nine participants were female and three were male (Table 2). Interview durations ranged from 40 to 60 minutes (mean 48 [SD 5.5] minutes). All individuals who had volunteered to participate in the study were interviewed, and study recruitment ended once the target of 12 completed telephone interviews had been reached.

**Table 2:** Participant demographics.

Participant	Sex	Age	Ethnicity	Employment Status
1	Male	65	White	Retired
2	Female	Missing	Missing	Working part time
3	Male	64	White	Working full time
4	Male	64	White	Working part time
5	Female	60	White	Working part time
6	Female	58	White	Working part time
7	Female	55	Missing	Working part time
8	Female	55	White	Working part time
9	Female	Missing	Missing	Working part time
10	Female	63	White	Unable to work
11	Female	57	White	Working full time
12	Female	65	White	Retired

### 3.2 Qualitative themes

Six themes were identified: previous physical activity behaviours and associated barriers; outcomes and impact of LiveWell Dorset experience; finding out about LiveWell Dorset; initial engagement with LiveWell Dorset; specific LiveWell Dorset support; and general LiveWell Dorset approach.

### 3.3 Previous physical activity behaviours and associated barriers

Reflecting back to the time immediately prior to their involvement with the LWD service, most participants reported that their PA levels had been well below the recommended 150 minutes of moderate intensity PA per week. Participants cited a number of barriers to PA participation that they had been facing, including long-term physical health conditions and injuries, mental health problems resulting from negative life circumstances such as bereavement and relocation, a lack of motivation to exercise alone, concerns about the health risks posed by exercise following an extended period of sedentarism, and limited time and energy due to work commitments:

*It was awful. I would leave in the morning at six thirty, work in a high-pressure job, often I would say at least a 60-h week. And you come back…let's say seven o'clock at night, and I'm going again at half past six in the morning, do I really want to go have a work out at the gym or do I want to go home? And so I tried to combat that by swimming, a kilometre, every lunch time at work… but actually some days I found myself falling asleep in my office, you know, the physical exertion was just too much.* [Participant 4]

### 3.4 Outcomes and impact of LiveWell Dorset experience

The majority of participants reported that LWD influenced them to make positive changes to their PA behaviour:

*So I've done loads of walking. I've done lots of YouTube exercise videos, I've done strength workouts, yoga, pilates, abs, all sorts of fitness.* [Participant 7]

Participants also showed evidence of a newfound ability to problem solve and self-manage their PA behaviour as a result of LWD's support:

*I do my [online] classes on the Tuesday evening and the Thursday evening. And in the morning I get up in the morning and I just put my gym kit on and I work in that gym kit so that I'm ready to go. And if I don't feel like it, just logging in and occasionally not turning the camera on straightaway and taking the first few steps. There's never been a night where I haven't done the full class.* [Participant 8]

In addition to changes relating to their PA behaviour, participants also frequently spoke about weight loss as an outcome of their experience with LWD, as well as improved physical and mental health.

### 3.5 Finding out about LiveWell Dorset

Participants reported first becoming aware of the LWD service through a number of different channels. For some, the source was a health care professional:

*[The nurse] said right, here's LiveWell Dorset, phone these people up, they will offer you some support. They gave me a card that said call these people, and I did. It was only a brief conversation as I recall.* [Participant 1]

Others reported finding out about LWD via Facebook, emails they received from their employer or the local Council, friends and community outreach events:

*The first [event] I saw [LiveWell Dorset] at was in [my local] library. It was like an exhibition of health, and LiveWell Dorset had a stand there and loads of leaflets, and some very friendly people that you were able to talk with and talk about the service. And I found it really quite informative. They were, you know, they were knowledgeable. They were very enthusiastic.* [Participant 4]

A number of participants did state that they felt LWD relied too much on online promotion, which might not always be the most effective means of reaching older adults. A number of additional promotional strategies were suggested, including advertising in community venues such as supermarkets and libraries, and involving past service-users as volunteer LWD ‘champions’. Aside from talking about *how* they found out about LWD, a number of participants also alluded to the timing, and *when* they found out about the service as being important to their subsequent engagement:

*I had been thinking for a long, long time about joining slimming clubs, and they put leaflets through the door and I would look at it and think about it and not do anything about it. So when it's suggested to you that [LiveWell Dorset] might help and [the physiotherapist] actually gave me a card to do something about it. It just caught me at the right time. A positive time to do it.* [Participant 5]

### 3.6 Initial engagement with LiveWell Dorset

Having become aware of LWD, participants identified a number of characteristics that initially made them want to engage with the service. For some, it was simply the name that they found appealing, and its association with wellbeing:

*I mean, LiveWell is a great title for what they do. You know, you don't need a psychology degree to work out LiveWell is about living better. It's great in that respect.* [Participant 2]

The local nature of the LWD service also appealed to some participants:

*It's good to know that there is something specific to Dorset that, yes, it's nice to know that there is specific help out there for anyone in Dorset that need it. It's just nice to know… It gives you a better feeling towards your community, knowing that there are others out there that want to use that service, that want to improve their lives.* [Participant 7]

Participants described registering to join LWD online or via telephone. Regardless of the mode, participants appreciated the straightforward, quick and flexible nature of the registration process. Some participants nonetheless mentioned the vulnerability they felt when taking the steps to register with LWD:

*It is daunting, it's really daunting. I think for anyone taking that first step, it's such a big thing. It's a really big thing. At the time I was actually feeling quite depressed as well. I was reaching out at a time when I felt vulnerable.* [Participant 2]

Following registration, most participants reported undergoing some form of lifestyle assessment with a LWD Wellness Advisor via telephone, which often had a motivational effect:

*I think I felt hopeful [following the assessment], which would have been the first time in a long while. She made me feel that there was something out there that could help me, whereas I'd been telling myself this was it, this is what I had and I had to put up with it and, you know, just get on with life as is.* [Participant 10]

As well as the free costs of the LWD service, a desire to repay the support and interest that LWD had shown them during their initial assessment also provided an incentive to move forward for some participants:

*It would be like, if you didn't [move forward from the assessment], it would be throwing it back at them. And if you can't be prepared to help yourself, then why should somebody else.* [Participant 5]

### 3.7 Specific LiveWell Dorset support

Participants accessed varying types and levels of LWD support. Following initial engagement with LWD, three received full coaching, three completed the Five Ways Challenge, three received low-level support in the form of follow-up phone calls or emails after their introductory assessment, and three did not receive any further intervention following their registration. All participants had access to the private LWD Facebook group and the array of online tools and apps.

Those participants who received coaching liked the self-directed nature of the coaching process:

*She didn't tell me what to do… She drew it out of me… So I was very open from the very moment and she was very like that counsellor in not telling me what to do, but in leading me, in guiding me.* [Participant 8]

Coaching participants also appreciated the practical solutions and suggestions that were frequently offered by their LWD Wellness Coach:

*She made it very much about her going away and coming back with some ideas for me as well. She came back with some links for me for doing exercises at home to cover the low cost. She told me about two outdoor gyms that are near me… My trainers weren't supporting me properly, and she said, go and buy yourself a new pair of trainers.* [Participant 8]

Participants who completed the Five Ways Challenge all felt that the structured nature of the programme had provided them with a sense of focus and routine:

*So it really just gave me a structure and a template to work towards of ideas and advice. But somehow by following a set programme… It's almost like you're being monitored. You're not being monitored but following a set programme makes all the difference* [Participant 7]

They also found that the weekly action planning worksheets that were supplied helped to support commitment and reflection:

*These sheets that you could print out… It was like a diary for me to fill in to show that I've actually achieved something each day, rather than just, you know, drifted and watched Netflix films… but also it makes you think in a positive way… I look back every now and again, and it's a little reminder of everything I've done.* [Participant 7]

Participants also found that the weekly Five Ways Challenge emails they received were helpful for providing ideas and trustworthy links to content:

*[The Five Ways Challenge emails] gave you information and ideas, you know? It was a lot easier because they were there. Whereas if you try and find [resources and information] by yourself and online, you can't always sort of get the right thing. Or you might have to pay for them. And they were there and I just have to click on each one and start… So that was, that's really useful.* [Participant 7]

They also liked the personalised nature of the emails, and the fact that they referred to the achievements of others involved in the programme:

*But the fact that there were lots of us doing it and different people were doing it. That was quite a nice feeling. I knew other people were having a go at different things. You know, they said, I don't know how many thousand people were doing it, and I thought, yeah, people are latching onto this. It's being part of something and doing it. Just knowing others were doing it.* [Participant 12]

The majority of participants reported viewing and/or using the private LWD Facebook group in some form. The peer support on offer was something that a number of participants appreciated:

*You couldn't pray for that level of support, you know? Bank that, you know, bottle it, it is just utter kindness. There's some real kindness in there. People really spurring each other on, and people are very open about what they're going through as well… It's a good group, really good group.* [Participant 6]

Some gained a sense of accountability and accomplishment from posting their own achievements and challenges for others in the group to see:

*I felt emboldened to actually post, why is it that I've gone up a dress size, and how on earth am I going to lose that? And I received some very encouraging responses. I think the fact that things are out there in the media as it were, it, it feels a bit more like a commitment. But by recording it, it endows it with a sense of accountability. It gives us that commitment, accountability, intent.* [Participant 9]

Participants also reported that they took inspiration from reading about the PA accomplishments of others in the Facebook group:

*I have to say other people inspired me on Facebook. They were happy. They showed that they were happy in themselves and, also, you know, they have the self-confidence to put themselves out there to show, show that they were pleased with their progress in what they did and what we could achieve… And it sort of inspired me to think.* [Participant 7]

Furthermore, some reported finding it rewarding to support others in improving their lifestyles:

*I have tried to chivvy people along. So I might go on and put something funny or pose a question. I've done that three or four times. Where people have had a particularly arduous journey, I want to congratulate people. But it just makes me feel part of that part of a community. It's a very real community.* [Participant 6]

A number of participants reported the usefulness of certain online tools and apps in aiding changes in their PA behaviour, such as the ‘Active Ten’ app, which automatically records brisk walking when the phone is placed in the pocket:

*The Active Ten app, which I picked up and had been using ever since… It just charts or logs as you walk. You have got to walk fairly briskly to jog it into action. And every ten minutes that you walk briskly, it rewards you with a shower of bunting and what have you, filling a challenge cup and it will chart up through the day how many of these cups you fill… Well I will deliberately set out to walk more briskly… It makes such a big difference… It's reinforcement.* [Participant 10]

### 3.8 General LiveWell Dorset approach

Regardless of the type of support accessed, a number of general characteristics of LWD's approach to influencing PA behaviour consistently attracted positive comments from participants. For instance, LWD's self-directed approach was often appreciated:

*I think most people would like to feel that they're doing it for themselves, even though they're being gently nudged in the right direction. I think that's what LiveWell Dorset is trying to do, which is great. It's not bossing people about and saying, for your health, you need to do this and this and this, it's gently nudging people in the right direction. And then they feel they're making the decisions themselves.* [Participant 12]

Also appreciated was LWD's empathy and kindness:

*They treat you like you're human, that you have feelings and that you are important. It's not like going to the doctors and you just get brushed off with a prescription, come back and see me in six weeks. There was a sort of a tough mother love there, but it was kindness and understanding and empathy. That made me feel comfortable. [Participant 10]*

LWD's non-judgemental approach was also often positively noted by participants:

*They're very non-judgmental. I think probably when you are trying to do something, achieve a healthy lifestyle goal… I guess a lot of people feel very judgmental of [their] own being, [their] own self. So you don't need anybody else to come along and judge you as well. You've done that quite enough already thank you very much. You've already made a judgment. You don't need somebody else to reinforce that in a sense. [Participant 9]*

LWD's focus on ‘small steps’ and breaking larger behavioural goals down into smaller chunks was also frequently appreciated by participants:

*It was absolutely achievable. And the fact that you could achieve it made you feel better. I think the fact that he just kept reminding us to just do little steps. Just do little steps. Don't worry about how far you're going. Just keep going forward. That's the most important thing. [Participant 11]*

A number of participants also found encouragement to be a valuable and motivational element of LWD's approach:

*The fact that there was somebody there just spurring me on, even though they didn't know me… They give you the little push to kind of say, yeah, it doesn't matter if you didn't do it today. Try again tomorrow kind of thing, rather than give up. So there was a lot in that. But there was somebody there and I think that's what has helped me tremendously. [Participant 12]*

Others appreciated the flexibility and individual tailoring. Finally, LWD's approach of offering an ‘open line’ for communication was also frequently valued by participants for the sense of security it provided:

*I think there was a phone number where if I was struggling at any time I could leave a message and they would get back to me. So they were always there in the background and knowing that I think helped as well. Just knowing that you have somebody… I just know having them there in the background was a comfort. [Participant 10]*

### 3.9 Behaviour change techniques

Fifteen different BCTs were identified as being used by LWD to facilitate participants’ progression through the service or to influence their PA behaviour. The most commonly identified BCTs were social support (unspecified) and adding objects to the environment. Table 3 provides an overall summary of the BCTs along with the components of the LWD service they related to. Definitions of the identified BCTs can be found in Supplement 1.

**Table 3:** Summary of behaviour change technique labels with related LiveWell Dorset components.

LiveWell Dorset Component	Behaviour Change Technique/s
Clinician/health care professional conveys information about LiveWell Dorset to individual	Social support (unspecified)
Clinician/health care professional conveys information on benefits of physical activity to individual	Information about health consequences
Clinician/health care professional provides card with information on how to register with LiveWell Dorset to individual	Social support (unspecified)
LiveWell Dorset runs stall at local health exhibition advertising LiveWell Dorset service	Social support (unspecified)
Information about LiveWell Dorset conveyed to individual via email from employer/council	Social support (unspecified)
Simple telephone and web-based sign-up processes provided for individual to register with LiveWell Dorset	Adding objects to the environment
LiveWell Dorset free to access	Restructuring the physical environment
Wellness Advisor conducts introductory LiveWell Dorset assessment with individual via telephone, completing and discussing current lifestyle and physical activity habits questionnaires with individual	Feedback on behaviour
Web-based ‘activity finder’ tool introduced to individual to help identify suitable physical activity opportunities to access locally	Adding objects to the environment
Phone-based ‘Active 10’ app introduced to individual to aid monitoring of physical activity	Adding objects to the environment
	Self-monitoring of behaviour
Open facility made available to individual to call or email LiveWell Dorset for support whenever required	Social support (unspecified)
Wellness Advisor conducts follow-up phone calls or sends follow-up emails to individual after introductory assessment	Social support (unspecified)
Wellness Coach provides general coaching support to individual via telephone	Social support (unspecified)
Wellness Coach provides practical support to individual during coaching	Social support (practical)
Wellness Coach sets physical activity goals with individual during coaching	Goal setting (behaviour)
Wellness Coach reviews physical activity goals with individual during coaching	Review behaviour goal(s)
Wellness Coach explores likely pros and cons with individual at end of 6 week coaching course of accessing another 6 week round of coaching	Comparative imagining of future outcomes
Wellness Coach provides Five Ways Challenge general communication and support to individual via email	Social support (unspecified)
Wellness Coach provides weekly Five Ways Challenge action template sheets for individual to plan activities	Adding objects to the environment
	Action planning
Wellness Coach provides weekly Five Ways Challenge worksheets for individual to record and review performed activities	Adding objects to the environment
	Self-monitoring of behaviour
Wellness Coach provides Five Ways Challenge information on available local and online activities to individual	Social support (unspecified)
Wellness Coach shares information about other's Five Ways Challenge achievements to individual	Social comparison
	Vicarious consequences
Online LiveWell Dorset community provides general support to individual via Facebook group	Social support (unspecified)
Individual able to share accomplishments with others in online LiveWell Dorset community	Focus on past success
Online LiveWell Dorset community provides information about other's achievements to individual	Social comparison
	Vicarious consequences
Individual able to provide support and encouragement based on their own experiences to others in online LiveWell Dorset community	Identity associated with changed behaviour

## 4 Discussion

Through gathering the views and experiences of individuals who had accessed LWD's support to increase their PA levels, it has been highlighted how the LWD service influences older adults' PA behaviour. This information is important, because with uncertainty around the most effective intervention characteristics and components to increase older adults' PA levels, there exists a need for further evidence in this area [7].

### 4.1 Principal findings

Despite initially facing a range of barriers to PA participation, participants reported that they had achieved increases in their PA levels and an improved ability to problem-solve and self-manage their PA behaviours as a result of their time with LWD, as well as noting impacts in other areas of their lives such as improved physical and mental health, and weight loss. In terms of how LWD influenced these changes, a number of key factors appear relevant.

Participants reported that LWD deployed a broad range of useful behaviour change strategies across the different types of support offered in order to facilitate their progress. These included providing diary worksheets for participants to plan and record their weekly activities as part of the Five Ways Challenge, and conducting goal-setting exercises during coaching sessions. The variety of strategies was also reflected in the BCT coding, which identified 15 different BCTs as being used by LWD to facilitate participants' progression through the service or to influence their PA behaviour. This finding supports previous reviews of literature on beneficial intervention components to increase the PA levels of older adults, where it has been established that behavioural and cognitive-focused behaviour change strategies are important elements of successful PA interventions [19,20], and that effective interventions typically incorporate greater numbers of BCTs, and utilise a blend of behavioural, motivational and/or cognitive methods to influence PA behaviour [7,19,21].

Participants also reported that LWD's approach to promoting initial awareness of the service through various channels, focus on opportunistically targeting individuals at key points in their life, and offering of a straightforward, quick and flexible registration process, helped to facilitate their initial engagement. As alluded to above, PA interventions, and related research, typically place a large emphasis on the ‘active ingredients’ of interventions that positively influence older adults' PA behaviour, such as BCTs and underpinning theoretical models [7]. However, with initial engagement representing the entry-point of participation, this finding highlights the equal importance of designing PA interventions that are accessible, so that they are eventually able to go on to influence PA behaviour. Previous reviews have also underlined this notion. The importance of looking beyond the behavioural and cognitive aspects of PA promotion and considering whole system-oriented approaches and the systemic and contextual factors and processes that encourage older adults' engagement with PA interventions has been highlighted, along with the need for future research to explore this area in greater detail [7,19,20]. A total absence of PA interventions for older adults co-created with input from their intended end-users has also previously been noted [7]. Including the intended recipients as stakeholders in the intervention development process, which LWD's operators seemingly did when they carried out consultations with local residents when designing their service, may be one method to ensure the most accessible interventions.

Aside from specific behaviour change strategies, participants also frequently reported that the social support offered to them by LWD was helpful, which included the ‘open line’ of email or telephone communication provided. Participants also reported that the social support offered by other service users through LWD was appreciated, such as through the LWD Facebook group. This finding again supports previous reviews that have reported that social contact and support are particularly important facilitators of PA participation in ageing populations [7,[22], [23], [24]]. In the most recent review, it was stated that emotional support from other people is linked with intrinsic motivation for PA, and consequently, participation in PA [22]. The review concluded that PA interventions for older adults should specifically seek to provide social support for individuals, which LWD appears to do in a variety of ways.

Closely aligned to the notion of social support, participants also reported that LWD's person-centred approach was helpful. For instance, participants valued LWD's encouragement of their autonomy and self-accountability, and also felt that the support they offered was tailored to their needs. In addition to person-centredness, the empathy at the heart of LWD's approach was also frequently appreciated. Supporting previous research, person-centredness, and in particular, tailoring has been noted as an important element of effective PA interventions for older adults, with robust evidence backing its value [7]. However, the finding that participants valued the empathy they received is more novel, with this concept less extensively researched for its role in influencing PA behaviour, and a need for further enquiry previously highlighted [7]. Long-standing evidence in the field of psychotherapy suggests that the levels of empathy displayed by professionals strongly predict positive patient outcomes in psychology-based treatments. This suggests that it is important that the role of empathy is directly examined in relation to the outcomes of PA interventions for older adults, given that like psychotherapeutic interventions, they aim to enact some form of healthy, adaptive behaviour, centred on interactions between professional and service-user, or ‘change agent’ and ‘change seeker’ [25,26].

### 4.2 Applications

With uncertainty around the most effective intervention characteristics and components to increase older adults' PA levels, there is a need for research exploring how different interventions influence older adults' PA behaviour [7]. The findings of this study provide useful information on how one particular intervention, LWD, appears to do so. This information can now potentially contribute to the understanding of ‘real-world’ public health practitioners on the needs and preferences of older adults as they attempt to change their PA behaviour, and on how to develop the components of interventions that they will willingly engage with. To further this understanding, the current findings also provide additional rationale for future research into previously highlighted topics of interest, such as the role of systemic and contextual factors and processes in encouraging older adults' initial engagement with PA interventions, and the role of professional empathy in PA intervention outcomes.

### 4.3 Limitations

Some limitations of this study should be noted. Firstly, the sample size was small, comprised of older adults from a rural geographical region and relatively narrow age range, which arguably limits the generalisability of the findings. Relatedly, a further limitation was the self-selection of interview participants. This may have related in some way to the behaviours, attributes and opinions being investigated, as well as to pre-existing levels of positivity towards LWD. Pragmatic factors limited the methods that were used to recruit participants, but it is fair to say that additional insights might have been gained with a less self-selected sample. For instance, a purposive sample of individuals that had been referred to LWD but didn't engage, or who engaged but dropped out, may have yielded very different and relevant information. Finally, content saturation was not reached during data collection. Resource constraints on the number of interviews that could be conducted, and the heterogeneous nature of participants' LWD experiences likely contributed to this. However, as data analysis partly involved deductive processes, with a priori codes derived from the original interview topic guide, it could be argued that as the pre-determined issues of interest were adequately represented in the data, the lack of traditional content saturation was not detrimental [27].

## 5 Conclusions

In this qualitative study a number of factors have been identified highlighting how the LWD service influences PA behaviour in older adults. These include using a broad range of promotional and behaviour change strategies to facilitate initial engagement, providing individuals with opportunities to receive social support from both professionals and peers, and emphasising person-centredness and empathy. Despite the limitations of this study, its findings provide valuable information to public health practitioners on some of the potential needs and preferences of older adults when attempting to influence their PA behaviour, and on reportedly helpful components of interventions that aim to do so. The findings also provide additional rationale for future research into previously highlighted areas of interest such as the roles of systemic and contextual factors and professional empathy on PA intervention engagement and outcomes.

## Contributors

The primary author conceptualised, designed and conducted this work, with assistance from the co-author. The primary author secured funding for this work from Active Dorset. The primary author prepared an initial draft paper, with the co-author contributing to subsequent drafts, and both authors approving the final manuscript.

## Funding

This work was supported by Active Dorset (grant number RED10671, 2018) via funding they initially received from Sport England. Active Dorset had no role in: data collection; data analysis and interpretation; and manuscript preparation, review and approval.

## Ethics approval

Ethical approval for this work was obtained from Bournemouth University (ref. 28034).

## Data sharing statement

No additional data are available.

## Declarations of competing interest

None.
