# Supplementary material for: How the LiveWell Dorset lifestyle behaviour change service influences older adults’ physical activity behaviour: A generic qualitative study
Source: Public Health Pract (Oxf). 2021 Jul 17;2:100164. doi: 10.1016/j.puhip.2021.100164 (PMC9461534; doi:10.1016/j.puhip.2021.100164)
Supplement: Multimedia component 1 [file mmc1.docx]

**Supplement 1 –** Definitions of behaviour change techniques.^17^

| **Behaviour Change Technique** | **Definition** |
| --- | --- |
| Social support (unspecified) | Advise on, arrange or provide social support (e.g. from friends, relatives, colleagues, buddies or staff) or non-contingent praise or reward for performance of the behaviour |
| Information about health consequences | Provide information (e.g. written, verbal, visual) about health consequences of performing the behaviour |
| Self-monitoring of behaviour | Establish a method for the person to monitor and record their behaviour(s) as part of a behaviour change strategy |
| Restructuring the physical environment | Change, or advise to change the physical environment in order to facilitate performance of the wanted behaviour or create barriers to the unwanted behaviours |
| Adding objects to the environment | Add objects to the environment in order to facilitate performance of the behaviour |
| Feedback on behaviour | Monitor and provide information or evaluative feedback on performance of the behaviour |
| Goal setting (behaviour) | Set or agree a goal defined in terms of the behaviour to be achieved |
| Action planning | Prompt detailed planning of performance of the behaviour |
| Social support (practical) | Advise on, arrange or provide practical help (e.g. from friends, relatives, colleagues, buddies or staff) for performance of the behaviour |
| Review behaviour goal(s) | Review behaviour goal(s) jointly with the person and consider modifying goal(s) or behaviour change strategy in light of achievement |
| Comparative imagining of future outcomes | Prompt or advise the imagining and comparing of future outcomes of changed versus unchanged behaviour |
| Social comparison | Draw attention to other’s performance to allow comparison with person’s own performance |
| Vicarious consequences | Prompt observation of the consequences (including rewards and punishments) for others when they perform the behaviour |
| Focus on past success | Advise to think about or list previous successes in performing the behaviour (or parts of it) |
| Identity associated with changed behaviour | Advise the person to construct a new self-identity as someone who ‘used to engage with the unwanted behaviour’ |
